# BUILDING COMMUNITY RELATIONSHIPS FOR MORE MEANINGFUL ENGAGEMENT WITH OLDER BLACK ADULTS

**DOI:** 10.1093/geroni/igad104.1461

**Published:** 2023-12-21

**Authors:** Patrice Fuller, Charles Fennell, Allison Lindauer, Raina Croff

**Affiliations:** Oregon Health & Science University, Portland, Oregon, United States; Oregon Health & Science University, Portland, Oregon, United States; Oregon Health & Science University, Portland, Oregon, United States; Oregon Health & Science University, Portland, Oregon, United States

## Abstract

Black people are grossly underrepresented in clinical trials, especially in Oregon, where only 2% of the population is Black. The Oregon Alzheimer’s Disease Research Center’s (OADRC) Outreach, Recruitment, and Engagement (ORE) Core aims to correct this disparity by promoting research participation, fostering retention, and supporting quality experiences for Black Oregonians. Essential to this goal has been having an ORE Core that is 50% Black, leaders experienced in Black-centered cognitive health research, and a team that demonstrates commitment to community partnerships. The ORE Core engages a Community Outreach Specialist (COS) whose efforts reflect our Core’s value of “service before sign-up.” The COS differs from a traditional “recruiter” in that the focus is on community relationship-building, rather than recruitment. This can only be supported by first serving the community (e.g., joining community boards, volunteering) before asking community members to sign-up for research. The OADRC promotes research participation by supporting studies that focus exclusively on older Black health. The African American Dementia and Aging Project, supported by a valuable community health partnership, aims to understand lifestyle factors affecting cognitive health. The Sharing History through Active Reminiscence and Photo-imagery Study (SHARP) aims to improve brain health through a culturally celebratory framework centering the Black experience. As a result, SHARP has sustained high retention rates and increased Black participation in Center research. We discuss the role of ORE Cores, the role, values, and reflections of our COS, and examine how Center studies foster sustained community relationships, ultimately for more meaningful engagement within the Black community.

